# Leveraging Artificial Intelligence for the Diagnosis of Systemic Sclerosis Associated Pulmonary Arterial Hypertension: Opportunities, Challenges, and Future Perspectives

**DOI:** 10.3390/arm93050047

**Published:** 2025-10-17

**Authors:** Samiksha Jain, Avneet Kaur, Abdul Qadeer, Victor Ghosh, Shivani Thota, Mallareddy Banala, Jieun Lee, Gayathri Yerrapragada, Poonguzhali Elangovan, Mohammed Naveed Shariff, Thangeswaran Natarajan, Jayarajasekaran Janarthanan, Jayavinamika Jayapradhaban Kala, Samuel Richard, Saai Poornima Vommi, Shiva Sankari Karuppiah, Anjani Muthyala, Vivek N. Iyer, Scott A. Helgeson, Dipankar Mitra, Shivaram P. Arunachalam

**Affiliations:** 1 Guntur Medical College, Guntur 522004, Andhra Pradesh, India; 2 Department of Internal Medicine, MedStar Union Memorial Hospital, Baltimore, MD 21218, USA; 3 Department of Cardiovascular Medicine, The University of Texas Medical Branch, Galveston, TX 77555, USA; aqkhan3611@gmail.com; 4 Andhra Medical College, Visakhapatnam 530002, Andhra Pradesh, India; victor.aryaghosh@gmail.com; 5 Kamineni Institute of Technology and Sciences, Hyderabad 508254, Telangana, India; shivanithota99@gmail.com; 6 Department of Radiology, University of Miami/Jackson Health System, Miami, FL 33136, USA; 7 Digital Engineering & Artificial Intelligence Laboratory (DEAL), Department of Critical Care Medicine, Mayo Clinic, Jacksonville, FL 32224, USA; lee.jieun@mayo.edu (J.L.); gayathriy9322@gmail.com (G.Y.); mohammednaveedshariff.r@gmail.com (M.N.S.); thangeswarann@gmail.com (T.N.); the.samuel.richard@gmail.com (S.R.); poornima3103@gmail.com (S.P.V.);; 8 Department of Cardiovascular Sciences, East Carolina University, Greenville, NC 27858, USA; 9 Division of Pulmonology, Department of Medicine, Mayo Clinic, Rochester, MN 55905, USA; 10 Division of Pulmonology & Department of Critical Care Medicine, Mayo Clinic, Jacksonville, FL 32224, USA; 11 Department of Computer Science & Computer Engineering, University of Wisconsin-La Crosse, La Crosse, WI 54601, USA

**Keywords:** systemic sclerosis, pulmonary arterial hypertension, artificial intelligence, machine learning, deep learning

## Abstract

**Highlights:**

**What are the main findings?**

- Artificial Intelligence (AI), particularly machine learning (ML) and deep learning (DL), shows significant potential in improving the early and non-invasive diagnosis of systemic sclerosis-associated pulmonary arterial hypertension (SSc-PAH).
- AI models can enhance the interpretation of a wide range of diagnostic modalities, including echocardiography (ECHO), ECGs, chest X-rays (CXRs), and biomarkers, often identifying subtle patterns missed by conventional analysis.
- Emerging SSc-PAH-specific AI applications successfully leverage genomics, proteomics, and multimodal clinical data for early detection, risk stratification, and prognostication.

**What is the implication of the main finding?**

- The integration of AI into screening algorithms could revolutionize SSc-PAH management by reducing diagnostic delays, optimizing referrals for right heart catheterization (RHC), and enabling personalized treatment strategies.
- Future, validated AI tools hold promise for deployment in diverse clinical settings, including resource-limited areas, to improve the poor prognosis associated with this life-threatening complication.

**Abstract:**

Systemic sclerosis-associated pulmonary arterial hypertension (SSc-PAH) is a life-threatening vascular complication of SSc, marked by high morbidity and mortality. Early diagnosis remains a major challenge due to nonspecific symptoms and the limitations of conventional tools such as echocardiography (ECHO), pulmonary function tests (PFTs), and serum biomarkers. This review evaluates the emerging role of artificial intelligence (AI), particularly machine learning (ML) and deep learning (DL), in improving the diagnostic landscape of SSc-PAH. A comprehensive literature search was conducted across PubMed, Scopus, IEEE Xplore, Embase and Google Scholar to identify studies involving AI applications in SSc, pulmonary arterial hypertension (PAH), and their intersection. Evidence indicates that AI models can assist interpretation across modalities, including heart sounds, ECGs, chest X-rays (CXRs), ECHOs, CT pulmonary angiography (CTPA), and omics-based biomarkers. While several models show encouraging diagnostic performance, their accuracy varies by dataset and modality, and most require external validation against right heart catheterization (RHC)-confirmed cohorts. Integrating multimodal data through AI frameworks may enhance early recognition and individualized risk stratification; however, these tools remain exploratory. Future work should emphasize harmonized hemodynamic definitions, transparent validation protocols, and SSc-specific datasets to ensure clinical applicability and reproducibility.

## 1. Introduction

Scleroderma, a term derived from “skleros” (hard) and “derma” (skin), or systemic sclerosis (SSc), is a rare, multifactorial condition affecting the connective tissue characterized by alterations in the blood vessel structure and functioning, extensive fibrosis, and the production of autoantibodies that are specific to different cell antigens [1,2]. The worldwide incidence of SSc ranges from 1.4 to 8.6 cases per 100,000 person-years, while its prevalence is estimated at 17.6 to 18.9 cases per 100,000 individuals [3]. Clinically, SSc is categorized into two types based on the degree of skin involvement, which include limited cutaneous SSc (lcSSc) and diffuse cutaneous SSc (dcSSc) [4]. In dcSSc, the skin thickening rapidly spreads from the skin distally to proximally, involving the arms, face, and trunk, and often involving the risk of lung fibrosis and renal crises. On the other hand, in lcSSc, skin involvement progresses gradually, affecting mainly the distal fingers, limbs, and face, avoiding the trunk. Raynaud’s phenomenon often precedes other manifestations in lcSSc by several years, and a subset of these patients may develop CREST syndrome (Calcinosis, Raynaud’s, Esophageal dysfunction, Sclerodactyly, and Telangiectasia), although it can also be seen in dcSSc [1,5]. The pathogenesis of SSc involves dysregulation of the immune system, vasculopathy, and fibroblast activation. Endothelial injury, mediated by cytokines like platelet-derived growth factor (PDGF), transforming growth factor-beta (TGF-β), and endothelin-1, plays a major role in vascular remodeling, capillary rarefaction, and the overproduction of collagen, leading to the fibrosis of skin and organs [6]. Among the systemic manifestations of SSc, pulmonary disorders are the leading cause of death [7]. Particularly, PAH, a serious vascular complication of SSc, is associated with a considerably high morbidity and mortality risk [8]. Approximately 8–12% of people with scleroderma develop PAH, which entails a 3-fold increase in the risk of death compared to patients without PAH [9]. SSc-PAH’s core pathogenesis is an obliterative vasculopathy that involves intimal proliferation, medial hyperplasia, and adventitial fibrosis. The reason for the initial trigger that leads to vascular involvement is still unknown, although it is considered to be an endothelial injury and dysfunction in some genetically predisposed individuals [7]. Early-stage SSc-PAH detection is a difficult clinical task that often needs to be differentiated from other diseases. Right heart catheterization (RHC) remains the gold standard for diagnosis, enabling unambiguous hemodynamic evaluation and excluding the misclassification of pulmonary hypertension subtypes [10,11]. On the contrary, along with its diagnostic accuracy, RHC also suffers from the drawbacks of being an invasive and expensive procedure that is adhered to by only a small number of patients [12]. Conversely, non-invasive diagnostic tools, like echocardiography (ECHO), pulmonary function tests (PFTs), and biomarkers, e.g., N-terminal pro-brain natriuretic peptide (NT-proBNP), are frequently used for screening. Nonetheless, these methods typically demonstrate lower specificity and sensitivity compared to RHC [12,13]. The clinical signs of PAH are mainly manifested with the presence of dyspnea, fatigue, exertional syncope, and symptoms of right heart dysfunction that are caused by the increased resistance of the pulmonary vasculature [10]. As of now, there are no such treatments that can completely cure the disease [14]. The primary goal of SSc-PAH therapy is to slow down the disease development and also enhance the quality of life of patients [13,15]. The contemporary pharmacological approaches comprise pulmonary vasodilator drugs that dilate pulmonary vessels through the targeting of different molecular pathways that control pulmonary vascular tone [15], restoring the affected endothelium by inhibiting the ET-1 action and increasing the prostacyclin (PGI2) and nitric oxide (NO) pathways [13]. Artificial intelligence (AI) is a very important subdivision of computer science that is involved in the process of making sophisticated machines that can perform human tasks [16]. AI is a helpful tool in the medical field, with applications in the diagnosis of diseases, the analysis of medical images, the proposal of treatment, the assessment of risks, and the optimization of healthcare. The latest developments in AI have made it possible for these machines to browse large databases, identify patterns, and even find solutions to different problems automatically, just with a little assistance from humans [17]. The different subdivisions of AI technology include computer vision, deep learning (DL), and machine learning (ML). Machine learning is the capacity of a computer program to adapt and develop continually from experience, which is very useful for the personalization of patient care and the adaptation of clinical decision-making. DL is a more advanced version of ML that is based on an artificial neural network (ANN) that acts like the human brain to simulate learning and making self-decisions [18]. Today, the field of AI in healthcare expands from general diagnostic instruments like ECG understanding to the discovery of intricate conditions like diabetic retinopathy [19,20]. Image analysis and real-time procedural guidance are the usage of AI in areas like Cardiology and Gastroenterology, which are procedure-oriented fields [21], whereas non-procedural sectors like endocrinology and rheumatology apply AI in solving diagnostic problems and treatment optimization [22]. Embedded with AI, wearable devices and smartphone apps have made it possible for patients to be monitored continuously and for doctors to be provided with real-time data to enable the timely detection of complications and manage chronic diseases more efficiently [17].

AI is already making tremendous progress in the healthcare sector, thereby enhancing clinicians’ efficiency and improving patient outcomes. However, sufficient information is required to assess their validity before regular use in clinical practice, and it is also essential to identify ethical and operational gaps that need to be bridged to achieve public trust and equity in access [23]. Although many studies have investigated the potential of AI in SSc and on AI applications in the traditional diagnostic methods of PAH, very few studies have addressed the joint use of these technologies, particularly in the case of SSc-PAH. This literature review evaluates the emerging role of AI, particularly ML and DL, in improving the diagnostic landscape of SSc-PAH. A comprehensive literature search was conducted across PubMed, Scopus, IEEE Xplore, Embase, and Google Scholar to identify studies involving AI applications in SSc, PAH, and their intersection. Building on these findings, we propose a conceptual framework for developing an exclusive, multimodal AI-based diagnostic strategy tailored for early recognition and individualized management of SSc-PAH. The review first highlights the clinical significance and diagnostic challenges of SSc-PAH, then provides an overview of relevant AI methodologies. We then synthesize applications of AI in diagnostic, prognostic, and risk stratification domains, and conclude by addressing current limitations and outlining future directions.

## 2. Materials and Methods

This work was conducted as a narrative review synthesizing the available literature on the application of AI in the diagnosis of SSc-PAH. The objective was to provide an integrated overview of current developments, methodological diversity, and potential translational pathways rather than perform a quantitative meta-analysis. These examples are intended to illustrate possible applications rather than serve as definitive evidence. The overall objective was to evaluate the evolving role of AI in SSc-PAH diagnosis and explore how existing models, particularly those developed for general PAH or SSc, might be adapted using disease-specific patient data to enable timely, accurate, and non-invasive diagnosis.

### 2.1. Search Strategy

A comprehensive literature search was carried out across PubMed, Scopus, IEEE Xplore, Embase, and Google Scholar for publications between 1999 and June 2025. Search terms included: “systemic sclerosis” OR “scleroderma” AND “pulmonary arterial hypertension” AND (“artificial intelligence” OR “machine learning” OR “deep learning” OR “biomarkers” OR “diagnostic imaging” OR “predictive modeling”). Reference lists from key review articles were also manually screened to capture additional relevant studies.

### 2.2. Selection and Scope

All studies that explored or discussed the role of AI, ML, or DL in SSc, PAH, or their intersection were considered. This included both peer-reviewed publications and conference abstracts presenting original data or conceptual frameworks.

### 2.3. Exclusion Criteria

Articles were excluded if they were non-English, animal or in vitro studies, or focused exclusively on therapeutic modeling without diagnostic or prognostic relevance.

### 2.4. Data Organization

The included literature was organized into three thematic domains:

1. AI in SSc;
2. AI applied to diagnostic modalities relevant to PAH (e.g., ECG, ECHO, CT, CMR, biomarkers);
3. Direct AI applications in SSc-PAH detection or risk stratification.

### 2.5. Appraisal of Evidence

Given the narrative design, no formal risk-of-bias or quality-assessment tool (e.g., PROBAST or QUADAS-2) was applied. However, studies were qualitatively reviewed for methodological soundness, transparency, and adherence to accepted hemodynamic definitions of PAH based on the 2022 ESC/ERS guidelines. Whenever available, external validation, sample size, and calibration details were noted.

### 2.6. Methodological Limitations

The review’s scope included both established and emerging studies, leading to variability in study design, dataset size, and validation rigor. Conference abstracts were included when they offered unique insights, though their limited peer review may affect reproducibility. Despite this heterogeneity, all data were cross-checked for internal consistency, accurate citation, and alignment with current clinical and hemodynamic standards.

## 3. Pathogenesis of SSc-PAH

SSc-PAH is primarily the result of a chronic remodeling of the pulmonary vasculature, typically obliterative pulmonary vasculopathy [7]. Endothelial dysfunction, immune dysregulation, and dysregulated intercellular communication cascades are some of the major mechanisms of the complex vasculopathy.

### 3.1. The Initial Vascular Insult: Endothelial Injury and Etiologic Agents

Recent evidence suggests that the pathogenic cascade in SSc-PAH is initiated by injury to, and resultant failure of, the pulmonary endothelial cells. The initial insult is of multifactorial etiology.

#### 3.1.1. Endothelial Cell Dysfunction

Endothelial damage by circulating factors such as anti-endothelial cell autoantibodies and other immune-activated factors usually found in SSc is one of the first events [7,24]. Disruption of endothelial integrity due to this is recognized as an important early process in disease development.

#### 3.1.2. Genetic Predisposition and Environmental Modulators

There exists a genetic predisposition to the development of SSc and its ensuing complications, including PAH. There are reported associations between certain Human Leukocyte Antigen (HLA) alleles and other genes implicated in immune response, vascular integrity, and fibrotic pathways that convey increased risk for SSc and PAH [7,25]. Moreover, environmental exposures like silica dust, solvents, and specific viral pathogens have also been recognized as modulators that are capable of triggering or exacerbating the inflammatory and fibrotic cascades that underlie SSc pathogenesis and the development of ensuing PAH [26,27].

### 3.2. Disruption of Vascular Homeostasis

Endothelial dysfunction deranges the delicate equilibrium between vasoconstrictive and vasodilatory signals in the pulmonary vasculature. This derangement results in an excess of powerful vasoconstrictors, particularly endothelin-1 (ET-1) and thromboxane A2, and a lack of generation or availability of critical vasodilators like NO and prostacyclin (PGI2) [7,25,28]. ET-1 not only causes severe vasoconstriction by activating ETA receptors but also induces vascular smooth muscle cell proliferation and migration, in part through an abnormal upregulation of ETB receptors typical of PAH [25,29,30,31]. Simultaneously, derangement of the NO-cyclic guanosine monophosphate (cGMP) and prostacyclin-cyclic adenosine monophosphate (cAMP) signaling pathways further aggravates vasoconstriction and deranges the counter-regulatory processes against cellular proliferation [25].

### 3.3. Immune System Dysregulation: A Key Pathogenic Factor

Immune system dysregulation is the defining feature in the pathogenesis of SSc-PAH and includes the involvement of both innate and adaptive immunity [32].

- Lymphocyte-Mediated Effects: T lymphocytes, particularly Th2 cells, are involved in fibrosis development through the release of cytokines such as interleukin-4 (IL-4) and IL-13 [14,33]. B lymphocytes participate in autoimmunity via the generation of autoantibodies (e.g., anti-endothelial cell antibodies) and in fibrotic processes by direct cell-to-cell interaction with vascular components [34,35].
- Role of Autoantibodies: Anti-topoisomerase IIα (anti-topo IIα) antibodies have been identified in a subset of SSc patients and are significantly associated with the development of PAH. Unlike anti-topoisomerase I or anti-centromere antibodies, anti-topo IIα is specifically linked to reduced diffusing capacity of the lung for carbon monoxide (DLco) and the presence of PH, independent of interstitial lung disease. This suggests a potential pathogenic role of anti-topo IIα in promoting vascular injury or dysfunction, contributing to the development of SSc-PH [36].

### 3.4. Pro-Remodeling Environment: Role of Growth Factors and Cytokines

Several growth factors and cytokines are involved in the vascular remodeling of SSc-PAH. Transforming Growth Factor-beta (TGF-β), the pivotal fibrogenic mediator, is present in higher levels in SSc and causes excessive deposition of extracellular matrix, thus leading to increased vascular stiffness and luminal narrowing [37]. PDGF is also involved in inducing proliferation and migration of fibroblasts and vascular smooth muscle cells, thus leading to increased intimal and medial thickening of the pulmonary arteries [38,39]. Notably, ET-1, being a vasoconstrictor, is also mitogenic for vascular smooth muscle cells, thus leading to remodeling [25].

### 3.5. Pathological Culmination: Vascular Remodeling and Its Structural Consequences

The combination of chronic endothelial dysfunction, chronic vasoconstriction, persistent immune activation, and dysregulated growth factor signaling eventually leads to the characteristic pulmonary vascular remodeling. Pathological features include intimal proliferation, medial hypertrophy due to smooth muscle cell proliferation, and adventitial fibrosis and inflammatory cell infiltration [7]. Less frequent than in idiopathic PAH, plexiform lesions occur in SSc-PAH [7]. Additionally, in situ pulmonary microthrombosis, secondary to endothelial dysfunction and procoagulant state, adds to progressive luminal obstruction [7]. In a minority of cases, the histopathological appearance may include overlapping features with pulmonary veno-occlusive disease (PVOD), a fibrotic occlusive disease of small pulmonary veins and venules, and a very dismal prognosis [7].

### 3.6. Hemodynamic Effects and Right Ventricular Adaptation

**Figure 1:**
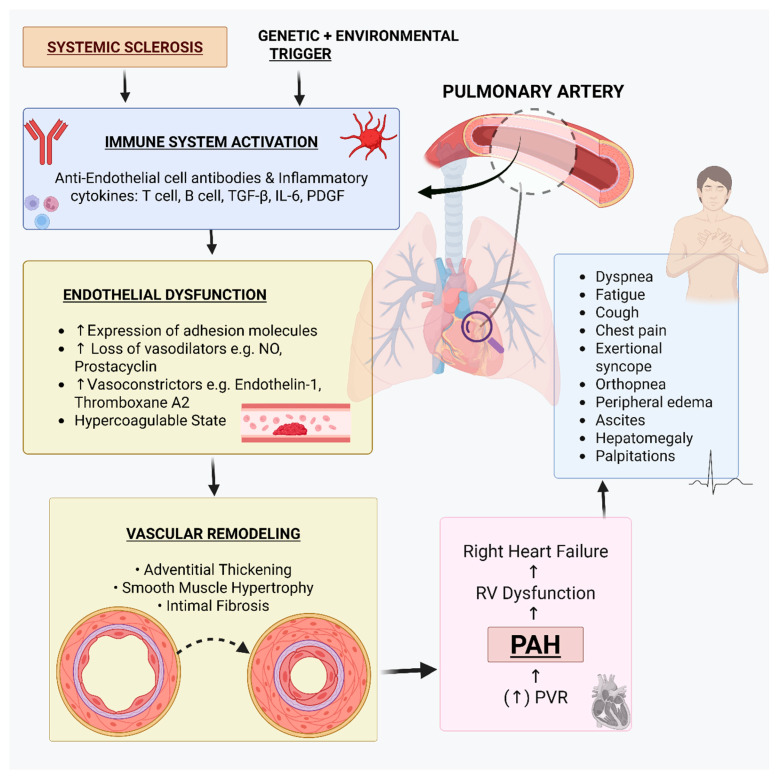
Proposed pathogenesis of SSc-PAH. ↑ indicates increased expression or activity. Boxes represent key pathogenic stages. Dashed circle highlights the pulmonary artery, showing a magnified cross-section.

These cumulative pathological alterations of the pulmonary vasculature result in a progressive, significant rise in pulmonary vascular resistance (PVR). To compensate for this chronically elevated afterload, the right ventricle (RV) initially adapts by hypertrophy to maintain cardiac output. A feature of SSc-PAH, however, is that the adaptational capacity of the RV is often impaired compared to that observed in idiopathic PAH. This can be explained by the direct involvement of the underlying SSc process (e.g., inflammation or fibrosis of the RV myocardium) or, perhaps, by the inherent biological variability of the RV of SSc patients [7,13,40,41]. The sustenance of severe pressure overload, coupled with this impaired RV adaptational capacity, ultimately results in RV dilation, progressive dysfunction, and overt right heart failure. The clinical symptoms of the patients with SSc-PAH are a consequence of the elevated PVR and resultant RV stress. Dyspnea on exertion is commonly the presenting and most frequent symptom, the failing capacity of the RV to augment pulmonary blood flow with increased physiological demand. With advancing disease, patients may develop severe fatigue, syncopal attacks (particularly on exertion), and atypical chest pain [42,43,44,45]. The onset of symptoms typical of advanced right-sided heart failure, like peripheral edema, ascites, and hepatomegaly, is an indicator of severe disease progression and is associated with an adverse prognosis [42,46]. Patients with SSc-PAH have a significantly worse prognosis compared to those with idiopathic PAH (IPAH) [47]. Figure 1 provides a visual representation of these pathogenic events, highlighting the key pathways and mediators involved.

## 4. Current Diagnoses of SSc-PAH

An accurate and timely SSc-PAH diagnosis is critical for initiating an appropriate and effective therapy. Differential diagnosis consists of a gradual and incremental distinction from other frequent causes of pulmonary hypertension (PH) in SSc patients, including PH caused by interstitial lung disease (ILD) (Group 3) and PH caused by left heart disease (Group 2). Thus, the optimal strategy is a combination of multimodal diagnostic strategies, integrating clinical evaluation, several non-invasive screening tests, and RHC [45,48].

### 4.1. The Need for Early and Accurate Detection

The typically insidious and non-specific nature of SSc-PAH symptoms in their initial presentation underscores the urgent necessity for early and accurate diagnosis. Early diagnosis is crucial for managing the natural history of the disease and maximizing long-term patient outcomes [49].

### 4.2. The Foundational Clinical Assessment

A thorough and systematic clinical evaluation forms the cornerstone of the diagnostic process in suspected SSc-PAH.

#### 4.2.1. Detailed Patient History and Review of Symptoms

A thorough patient history, with specific attention to PAH-suggestive symptoms and RV dysfunction and exercise intolerance, serves as the foundation for diagnosis [50,51]. Symptoms are the character, timing, and course of exertional dyspnea, unexplained fatigue, pre-syncopal or syncopal attacks, atypical chest pain, and peripheral edema or ascites. SSc-specific clinical features, general disease duration, and the patient’s autoantibody profile are all significant contextual information [36,42]. For instance, anti-centromere antibody-positive patients are at high risk of developing PAH [52]. One must also proactively inquire and cautiously consider symptoms that may suggest alternative or coexistent diagnoses, such as ILD, or if experiencing orthopnea and paroxysmal nocturnal dyspnea (typical of left heart disease) [48,53].

#### 4.2.2. Physical Examination Findings and Clinical Clues

While the physical examination is normal in early SSc-PAH, advanced disease later will reveal findings consistent with RV strain and increased pulmonary pressures. The most valuable findings are tachycardia, an accentuated P2, palpable RV heave or lift, tricuspid regurgitation murmur, dependent edema, or elevated JVP in advanced stages [10,54]. Recognition of findings of advanced RV failure, such as hepatomegaly, ascites, and pitting peripheral edema, signifies a more advanced stage of disease [42]. A thorough SSc-specific systemic examination, as well as careful attention to clinical signs of comorbid ILD or left heart disease, is also an essential component of the complete clinical assessment [48].

### 4.3. Diagnostic Armamentarium: Modalities Needed and How to Use Them

A sophisticated array of diagnostic studies is used to confirm clinical hypotheses, accurately define the hemodynamic disturbance, and guide therapeutic interventions in SSc-PAH.

#### 4.3.1. RHC: The Hemodynamic Gold Standard

RHC remains the gold standard necessary for the absolute diagnosis and global hemodynamic characterization of PAH. The invasive test provides immediate and precise measurements of the most relevant hemodynamic parameters, including mean pulmonary arterial pressure (mPAP), pulmonary artery wedge pressure (PAWP), PVR, and cardiac output. These values allow for the absolute diagnosis of pre-capillary PAH (as classified by mPAP > 20 mmHg, PAWP ≤ 15 mmHg, and PVR ≥ 2 Wood Units) and, importantly, the exclusion of post-capillary PH (Group 2 PH, PAWP > 15 mmHg) and mPAP >20 mmHg) [51,55,56]. RHC is not only necessary for diagnosis but also for the assessment of disease severity, vasoreactivity testing in selected patients (though less commonly performed in the SSc-PAH context), and decision-making for critical therapy [51,55,57]. However, its inherent invasiveness and requirement for specialized centers and skilled operators make it inappropriate as a screening test in the general SSc population [49].

#### 4.3.2. ECHO: The First Non-Invasive Imaging Modality

Transthoracic echocardiography (TTE) is recommended as the key non-invasive tool for screening and assessing the probability of PH in patients with SSc. The peak tricuspid regurgitation velocity (TRV) is the main variable used to estimate the echocardiographic probability of PH, rather than the calculated systolic pulmonary artery pressure (SPAP). A TRV > 2.8 m/s, together with additional echocardiographic signs such as right atrium (RA) or RV enlargement, interventricular septal flattening, or inferior vena cava dilation, indicates an intermediate or high probability of PH. RHC is required to confirm the diagnosis [51].

#### 4.3.3. PFTs: Indirect Vascular Disease Markers

PFTs, including spirometry with lung volumes and diffusing capacity of the lung for carbon monoxide measurement, are standard components of the overall evaluation of SSc patients [58]. PFTs play a vital role in SSc care by not only diagnosing ILD but also detecting early signs of pulmonary vascular disease. Studies show that a markedly reduced DLco (DLco < 60% predicted), or forced vital capacity(FVC)/DLco ratio (>1.6) strongly suggests pulmonary vascular involvement [55]. Furthermore, a consistently diminishing DLco, as clinically noted on serial testing, has been found to be a sensitive predictor of imminent PAH development [59]. However, an abnormal DLco is not specific to PAH and may occur in numerous other diseases, e.g., ILD itself; conversely, a small subset of SSc-PAH patients may have a normal DLco [60,61].

#### 4.3.4. Serum Biomarkers: Adjunctive Information for Risk Stratification

Serum biomarkers are valuable adjunctive aids to risk stratification and prognosis in SSc-PAH but are not individually diagnostic. Natriuretic peptides, such as brain natriuretic peptide (BNP) or NT-proBNP, are secreted in response to ventricular wall stress by the myocardial cells corresponding to hemodynamic severity and prognosis [51,62,63]. Elevated serum uric acid is also associated with PAH severity and adverse prognosis in SSc [64]. Red cell distribution width (RDW), a measure of variability in erythrocyte size readily available from a complete blood count, is generally elevated in SSc-PAH and potentially of prognostic value [65]. Other potential biomarkers under study are inflammatory, endothelial, or fibrotic markers (e.g., soluble suppression of tumorigenicity 2 [sST2], receptor for advanced glycation end products [RAGE], T-cell subsets, adipsin, and Complement Factor D), but they require validation [14,66,67,68,69]. While helpful, biomarkers generally lack specificity to diagnose PAH in isolation and are best interpreted within the context of clinical and imaging data.

#### 4.3.5. Exercise Testing

Measurement of exercise capacity is useful in functional impairment assessment and prognosis. The 6-min walk test (6MWT) is a simple, standardized submaximal test widely used in PAH trials and daily clinical practice. Prognostic data are provided by measurements like walking distance (e.g., <165 m predicts a high risk) and oxygen desaturation during the test. Cardiopulmonary testing with maximal exercise (e.g., measurement of peak oxygen consumption [VO_2_peak] and minute ventilation/carbon dioxide production [VE/VCO_2_] slope) is a more sensitive cardiopulmonary physiology evaluation but is not used more frequently for routine screening. Both tests measure exercise limitation, a predominant feature of symptomatic PAH [51,70,71,72].

#### 4.3.6. Chest-Xray, CT Pulmonary Angiography, and High-Resolution CT

Though chest radiography is insensitive to early PAH, it can detect cardiomegaly or enlarged central pulmonary arteries in advanced illness [73]. High-Resolution CT (HRCT) is essential in SSc-PAH for diagnosing and quantifying ILD and can also reveal features suggestive of pulmonary veno-occlusive disease (PVOD), such as interlobular septal thickening and ground-glass opacities. These findings help distinguish PAH subtypes and guide safer treatment decisions [74]. CT Pulmonary Angiography (CTPA) is held in reserve to be performed with V/Q scanning to rule out chronic thromboembolic pulmonary hypertension (CTEPH, Group 4 PH) [51,75]. CTPA can also provide supportive findings for PH through measurements such as main PA diameter (>30 mm) or PA diameter to aortic diameter ratio (>0.9) or ratio of segmental artery to bronchus in three or four lobes (>1:1), which are hemodynamically and prognostically significant [51,76,77].

#### 4.3.7. Cardiac Magnetic Resonance

Cardiac magnetic resonance imaging (CMR) is the international non-invasive gold standard for the evaluation of RV structure and function, of maximal importance in SSc-PAH. CMR permits accurate and reproducible quantitative evaluation of RV volumes (end-diastolic volume, stroke volume), mass, and ejection fraction (EF). CMR measurements are not confounded by acoustic windows. CMR also permits evaluation of interventricular septum morphology, and bowing towards the left ventricle is an indicator of RV pressure overload. CMR also permits characterization of myocardial tissue with modalities like late gadolinium enhancement (LGE) to identify areas of fibrosis or inflammation in the RV myocardium. Degree and extent of RV LGE on CMR may be of prognostic significance in PAH. CMR also permits assessment of pulmonary artery distensibility and blood flow dynamics, yielding further information regarding pulmonary vascular disease severity. Even though not commonly used in the first instance as a screening test due to cost and availability issues, CMR is highly useful in precise RV analysis and in monitoring disease course and treatment response, particularly when ECHO image quality is poor or precise quantification of RV function and structural change is necessary [78,79].

Given the limitations of individual tests, current practice advocates now advise the utilization of structured screening algorithms that combine clinical risk factors with the findings of non-invasive testing for systematic screening of SSc patients for high-risk candidates for PAH who are worthy of confirmatory RHC. This will serve to optimize early detection by optimizing the utilization of the invasive RHC procedure [80,81].

### 4.4. Screening Algorithms

#### 4.4.1. DETECT Algorithm

The DETECT algorithm is a two-step, evidence-based screening strategy established and validated for PAH detection specifically in SSc patients. The DETECT algorithm, derived from a large multicenter study, has been established to improve the timely detection of those patients requiring RHC. The DETECT algorithm consists of two steps. Step 1 contains non-echocardiographic variables: FVC% predicted/DLco% predicted, ACA presence, telangiectasias history, right axis deviation on ECG, serum value of NT-proBNP, and serum value of uric acid. The risk score is evolved based on these six variables. If the score is above a pre-specified cutoff score, the patient is referred to Step 2. Step 2 consists of echocardiographic parameters: TRV and RA area. Based on the integration of the risk score in Step 1 and the echocardiographic parameters in Step 2 (i.e., TRV > 2.8 m/s or TRV 2.5–2.8 m/s and RA area > 18 cm^2^), the algorithm recommends referral to RHC. The DETECT algorithm has been reported to have better sensitivity for the detection of SSc-PAH compared to traditionally used ECHO-based screening strategies alone, although perhaps with reduced specificity [80,82].

#### 4.4.2. ASIG Algorithm

The Australian Scleroderma Interest Group (ASIG) algorithm is yet another algorithm that has been put forward for screening for SSc-PAH that prioritizes simplicity and user-friendliness for use in everyday clinical practice. The ASIG algorithm consists of components such as PFTs (assessment of the DLco% predicted with FVC/DLco ratio) and serum NT-proBNP. A positive screen in the ASIG algorithm is either an FVC/DLco ratio ≥ 1.8 with DLco < 70% predicted or a serum NT-proBNP ≥ 210 pg/mL. Positive screening by either criterion should be referred for further evaluation, which may include ECHO and finally RHC if indicated [80]. Although less complex in design than DETECT, comparative studies suggest that the ASIG algorithm is also of reasonable sensitivity for SSc-PAH detection and may have better specificity for DETECT in certain patient types [80].

These systematic screening algorithms, through the incorporation of easily accessible clinical and non-invasive information, seek to optimize the early identification of SSc-PAH, enabling timely referral for confirmatory RHC and early institution of disease-directed treatments, ultimately with the goal of optimizing the poor prognosis of this serious complication.

## 5. AI in Healthcare

Artificial intelligence (AI) is a subdivision of computer science focused on developing intelligent systems capable of learning and decision-making [16]. In healthcare, AI, particularly machine learning (ML) and deep learning (DL), is increasingly used to analyze large datasets, identify hidden patterns, and support diagnostic and prognostic decision-making [18]. ML enables systems to learn from structured datasets and predict outcomes through methods such as supervised, unsupervised, and reinforcement learning [23,83,84]. DL, through neural network architectures such as convolutional neural networks (CNNs), enhances these capabilities by automatically extracting complex features from raw data [85,86]. In medicine, AI has shown strong performance in tasks such as medical imaging, ECG interpretation, and automated echocardiographic analysis, often detecting subtle abnormalities that may be missed by human review [87,88,89]. Its integration with digital health technologies, including wearable devices and EHRs, further enables continuous monitoring and rapid data analysis [90,91]. For SSc-PAH, the relevance of these advances lies not in generic healthcare applications but in their potential to be adapted for early detection of pulmonary vascular changes, improving sensitivity beyond current screening modalities and reducing reliance on invasive testing.

## 6. AI in SSc Diagnostics: A General Overview

The combination of AI and ML is rapidly revolutionizing the diagnostic methods in different medical fields, with a special focus on rheumatology [92]. Some of the applications of AI in SSc are as follows.

### 6.1. AI Applications in Comprehensive Scleroderma Evaluation System

AI is increasingly being used to study complex datasets in SSc with a perspective towards earlier diagnosis, improved disease stratification, and organ-specific prediction of involvement. Key applications include the following.

#### 6.1.1. Microvascular Assessment (Nailfold Videocapillaroscopy—NVC)

AI, particularly CNNs, is augmenting the objectivity and speed of NVC image analysis. DL algorithms are learned to detect and measure capillaroscopic features of SSc (e.g., giant capillaries, hemorrhages, avascular areas) [93,94,95,96]. CNN-based semantic segmentation can label and distinguish individual capillaries, which can be utilized to segment SSc-typical NVC patterns.

#### 6.1.2. Pulmonary Fibrosis (Interstitial Lung Disease—ILD) Quantification

Based on the extent of SSc-ILD, automated segmentation and quantification of fibrotic patterns (ground-glass opacities, reticulation, honeycombing) on HRCT images are being created with the aid of AI tools, in the form of the majority of CNNs (such as U-Net models) [97,98,99,100,101]. Quantitative HRCT (qHRCT) algorithms provide quantitative ILD severity scores and can enhance sensitivity in the monitoring of disease progression or therapeutic response.

#### 6.1.3. Skin Fibrosis Assessment

Objective assessment of skin fibrosis remains an issue in the present era. AI studies include the application of ML algorithms to high-frequency ultrasound image data for determining dermal thickness and echogenicity [102,103].

#### 6.1.4. Cardiac Involvement Detection

AI is emerging as a valuable tool in identifying cardiovascular complications in SSc. It is being used in retinal and cardiac imaging to detect early vascular changes and coronary artery calcification, aiding in timely risk assessment. DL techniques support tissue segmentation and fibrosis detection, particularly relevant to arrhythmia risk. Algorithms analyzing motion and deformation patterns, especially in the complex RV, are enabling earlier detection of subtle abnormalities. Additionally, models integrating clinical and imaging data are helping to guide treatment selection and predict therapeutic response, paving the way for more precise and proactive cardiac care in SSc [104,105].

#### 6.1.5. Disease Stratification and Phenotyping

To counter SSc heterogeneity, supervised (e.g., SVMs, Random Forests (RFs)) and unsupervised (e.g., clustering algorithms) ML approaches are used on large, multi-modal registries (imaging, genetic, serological, clinical) such as EUSTAR. This is to define discrete patient phenotypes with common pathobiology or prognostic courses to enable personalized medicine [106,107].

#### 6.1.6. Predicting Future Organ Involvement

ML-driven tools are being created to predict future organ involvement and complications (e.g., renal crisis, severe gastrointestinal issues) by utilizing parameters available at the beginning point [108].

The extraordinary impact of AI and ML in multiple organ systems in SSc stands as a testament to the efficacy of technology in dealing with the complicated and diverse nature of the disease. These breakthroughs not only validate the feasibility of AI integration in clinical practice but also serve as the initial step in its expansion into unexplored fields, for example, SSc-PAH, where early diagnosis and improved patient monitoring are of utmost importance. Following the successful use of these organ-specific models, implementing AI tools in SSc-PAH is a logical and promising next step.

## 7. Application of AI in Existing Traditional Diagnostic Modalities for SSc-PAH

The complexity and heterogeneity of SSc-PAH challenge early diagnosis through conventional methods. Currently, the commonly used screening tools for SSc-PAH, such as TTE, DLCO, and NT-proBNP, have limited sensitivity when used alone. The most routinely used tool, TTE, provides only an estimate of the PASP, with a sensitivity of 0.88, resulting in more false negatives and delayed diagnosis, and its specificity of 0.56 leads to more false positives and hence unnecessary RHC procedures [109]. Although RHC is the gold standard, its invasive nature, high cost, and limited availability make it unsuitable for becoming a large-scale screening tool. To overcome these gaps and challenges for the early detection of PAH in scleroderma patients, AI has emerged as an affordable screening tool with a higher accuracy than these conventional methods. From its evolution from a single-modal dataset to multimodal dataset integration, AI has the potential to simultaneously analyze the PFTs, various parameters of ECHO, lab markers, and electronic health records (EHRs) to predict the risk [110]. This section explores the applications of AI across various traditional diagnostic modalities, each with its unique findings.

### 7.1. AI in Non-Invasive Physiological Monitoring

#### 7.1.1. AI in Heart Sound Analysis

The process of auscultating heart sounds is a component of the standard clinical examination, but it does have limitations due to differences in clinical expertise as well as human hearing interpretations. Stethoscope recording applications have completely transformed with the now available digital stethoscopes and AI-enhanced auscultation devices. Ogawa et al. proposed a new technology called ‘Super Stethoscope’, which is a combination of a bioacoustics system and AI [111]. The device is equipped with ECHO and heart sound data that are both collected simultaneously, optimized for a signal-to-noise ratio from audible as well as inaudible frequency bands. Thus, it can detect subtle acoustic signs that can be missed by traditional stethoscopes. Furthermore, it also provides quantitative visualizations of heart sounds in clear representations, such as graphs or waveforms, that make it easier for clinicians to understand, even during remote auscultations. This device can also be used in PAH since it can pick up the extra heart sounds corresponding to the increased pulmonary artery pressures (PAPs), for example, loud P2 or right-sided murmurs, which makes it a potential new tool for early diagnosis and risk stratification of the condition. With the help of this device, the remote monitoring of patients suffering from PAH can be made more accessible, thus ensuring easy accessibility and continuity of care. The recent findings by Guo et al. introduced the use of DL-based models that use phonocardiogram (PCG) data to detect an elevated PASP, which is a critical indicator of PH [112]. The semi-supervised methodology used for training the model involved 6000 PCG recordings along with their respective ECHO-based PASP values and 169,000 PCGs without ECHOs. Digital stethoscope recordings captured PCGs and were then analyzed through 5-s mel-spectrograms for processing. The AI model tested here had a sensitivity of 0.71 and a specificity of 0.73, with the best performance in the pulmonic area and left subclavicular regions. Furthermore, with its GradCAM++ technique, physiologically relevant segments of heart sounds can be noticed in the PCG segment, highlighting the strong clinical focus of the model. AI-driven analysis of heart sounds can serve as a cost-effective, non-invasive, easily accessible screening tool for early PH detection. This approach, in its application to group 1 PH (PAH), without the need for other imaging tests such as ECHO or Cardiac MRI, can detect early changes in the vascular structure. Hence is a useful tool in settings where resources are scarce as well as for regular monitoring of the condition. Some other investigations have shown that AI models can facilitate the analysis of auscultated heart sounds and can aid in the classification of heart conditions such as PH. Elgendi et al. and Wang et al. presented excellent AI-powered frameworks for auscultation in the PH detection process [113,114]. SSc-PAH is a rare and fatal type of PH, so modifying these models to this specific group has great potential as an early, non-invasive, and scalable screening approach, especially in conjunction with clinical risk algorithms and telehealth technologies [113,114].

#### 7.1.2. AI in Biomarkers

BNP and NT-proBNP are the common biomarkers that are used in conditions such as acute or chronic heart failure [115,116]. NT-proBNP is also used to screen for PAH in patients with systemic sclerosis (SSc) and can also be a crucial biomarker in predicting mortality in PAH-CHD (congenital heart disease) patients [82,117]. Although NT-proBNP remains a clinically valuable marker, its interpretation continues to be difficult due to many factors, including the variability of levels in different patient populations and comorbid conditions. Hence, AI can be introduced here as a solution. Lewis et al., in their review, state that ML models can automatically process NT-proBNP levels, quickly analyze them with other clinical markers, give personalized treatment strategies, predict disease progression, and flag high-risk patients [118]. Routine measurement of lab-based assays for BNP and NT-proBNP uses venous blood samples, and the results are not available immediately. In contrast, AI-driven models can involve the development of point-of-care testing (POCT) platforms that can ensure immediate availability of the results, which can help in informed decision-making at the time of consultation itself. With the expanding role of AI integration with NT-proBNP, it holds a promising potential to improve prognostic assessments and optimize treatment plans.

#### 7.1.3. AI in ECG Interpretation

Expanding from the promising potential shown by the PFTs and CTPA models, Kwon et al. revealed that even a simple, widely available ECG, when interpreted by AI, can become a powerful, low-cost, non-invasive screening tool, especially in low-resource settings [119]. By utilizing a large dataset, the model was taught to identify the ECG patterns that are typically found in subjects with PH, hence the configuration of this design. The focus was on the minute changes in ECG wave pattern analysis (S/P/T waves), which are sometimes missed by doctors. Notably, the tool showed a higher accuracy in predicting patients at higher risk of PH even before the symptom onset. Although the model was created to detect PH broadly, its high predictive performance allows it to serve as the base for future PAH-specific applications. Because PAH is a subtype of PH and shares certain common ECG findings during the initial stages, like RA enlargement and RV strain. AI-ECG tools like this hold promise for future adaptation and validation in PAH-specific populations, offering a potential pathway for earlier intervention in PAH. Liu et al. (2022) and DuBrock et al. (2024) proposed highly efficient DL models based on high-accuracy ECG findings for the detection of PH. Liu’s model assessed 10 AI algorithms employing 12-lead ECG paired with ECHO estimates of PAP and presented high predictive capacity [120]. DuBrock’s PH Early Detection Algorithm (PH-EDA) utilized CNNs trained on ECGs from PH patients and was able to detect PH five years before diagnosis, even when ECGs were visually normal [121]. By fine-tuning these algorithms with SSc-specific ECG datasets and integrating longitudinal ECG changes, they could enable non-invasive, cost-effective screening and early risk stratification in high-risk SSc populations.

#### 7.1.4. AI in PFTs

Often overlooked as something minor, the PFTs of a patient with SSc can reveal subtle physiological changes that may point toward early vascular involvement before any symptoms or imaging abnormalities are detected. Schreiber et al. proposed a formula using SpO_2_ and DLco as parameters to predict the mean PAP (mPAP) as mPAP = 136 − SpO_2_ − 0.25 × DLco% in patients with SSc [122]. Although this formula was developed to detect all different forms of PH (including Groups 1–5), it has been particularly successful in the PAH (Group 1 PH) subgroup, where it reached 90% sensitivity for detecting patients who required RHC. If similar clinical variables are integrated into AI-driven models alongside PAH-specific markers like NT-proBNP and echocardiographic data, they could provide a promising alternative for early, non-invasive SSc-PAH detection without necessitating the direct involvement of a specialist. This will enhance the predictive accuracy of PAH in SSc patients by analyzing large datasets and, therefore, enabling a real-time risk assessment of individual patients.

#### 7.1.5. AI-Enabled Wearable Technology

Wearable devices combined with AI allow continuous, real-time monitoring of physiological parameters. These tools can provide clinically meaningful insights by detecting early functional decline outside of traditional hospital settings. Studies have shown the feasibility of using wearables to track activity and sleep patterns in patients with pulmonary hypertension [123]. Other technologies, such as wireless epidermal electronic systems (EEsS), can record ECG and seismocardiogram (SCG) signals, transmitting them to AI algorithms for the extraction of prognostic markers [124]. In SSc-PAH, where early symptoms such as reduced exercise tolerance are subtle and progressive, AI-enhanced wearables could provide longitudinal monitoring that complements structured screening algorithms. By integrating trends from daily activity or cardiopulmonary signals, such systems may help identify high-risk patients earlier, guide timely referrals for confirmatory testing, and support ongoing disease management.

#### 7.1.6. AI in EHRs

The application of AI to EHRs has unveiled new possibilities for disease prediction and timely diagnosis. Specialized training AI models, such as Med-BERT, have been used to analyze the EHR [125]. Trained on over 28 million patients, this model is based on the bidirectional encoder representations from the transformers (BERTs) framework, which allows it to understand the sequential and coded nature of the medical data. It can note complex patterns of diagnoses and procedures within these codes, even when the data available is very limited. Studies have shown that using Med-BERT in newer prediction models can improve their accuracy, even if they are trained on small datasets. AI technology has also paved the way for the detection of PAH in EHRs. Schuler et al. created an ML algorithm that recognized the cases independently based on the International Classification of Diseases (ICD) codes, diagnostic procedures, and medication data, which eventually led to the identification of patients with concealed PAH characteristics more accurately and with greater sensitivity than before [126].

### 7.2. AI-Driven Imaging-Based Diagnostic Approaches

#### 7.2.1. AI in CXRs

Aside from the aforementioned methods, CXR is a tool that is very frequently utilized and also has an AI-assisted application in the diagnosis of PH. Kusunose et al. employed a DL model for pre- and post-exercise CXR images of SSc or mixed connective tissue disorder to achieve exercise-induced pulmonary hypertension (EIPH) with high accuracy [127]. The early pulmonary vascular changes that this model identifies indicate that it could have the potential to be useful in the screening of high-risk populations for PAH in clinical settings where detailed modalities like stress ECHO are not easily accessible. Hence, it can enable doctors to perform early screening of PAH, even in places that do not offer invasive testing or have a specialist to interpret cardiac imaging. Imai et al. utilized a CNN model in CXR, pretrained on ImageNet-1K, to detect PAH [128]. The model performed better than a cohort of nine skilled respirologists and radiologists and exhibited sensitivity, specificity, and Area Under the Curve (AUC) values of 93.3%, 98.2%, and 0.988, respectively. A very important feature of the research was the use of RHC-confirmed cases, which consequently increased diagnostic reliability. Because of its great performance, this model has a breakthrough possibility to be improved through the incorporation of CXR datasets from SSc patients with and without PAH, enabling non-invasive and early detection of SSc-PAH. Zou et al. also demonstrated the capability of AI in CXR by accurately classifying the PH case from normal CXRs [129]. However, the model had been extrapolated from TTE estimates, which include PASP, instead of being based on direct hemodynamic measurements, which means that the model can be potentially biased, as these measurements can be altered by variations in the cardiac rhythm, patient positioning, and the operator’s skill. Consequently, the training or fine-tuning of these models is required to improve credibility and practical application in SSc-PAH.

#### 7.2.2. AI in ECHO

Salehi et al. evaluated the efficacy of the US2.AI tool, which incorporates a DL framework for automatic measurement of tricuspid regurgitation jet velocity (TRJV), which can estimate the pressure gradient between the RV and RA and help clinicians infer an elevated PAP pressure [130]. This AI tool was able to successfully interpret TRJV in about 87% of the cases that had already undergone both ECHO and RHC. Furthermore, it showed a high diagnostic accuracy for detecting PH in both its manual and automated approaches. The integration of AI in the echocardiographic evaluation, if achieved, can be fully automated, non-invasive, timesaving, and more accurate than the manual measurement, which will dramatically minimize the need for a skilled individual. The continuous monitoring of TRJV in this manner can be an early indicator of PH, thus allowing timely interventions and improved treatment results. For several decades, ECHO has been serving as a screening tool for PH, yet traditional ECHO analysis fails to identify early signs and provide an accurate estimate of PAP. A recent study by Liao et al. used ML models to evaluate ECHO for diagnosing PH [131]. The model was tested on a cohort of 346 patients with suspected PH who had already undergone RHC, which is the gold standard for confirmation. Images from their ECHO were collected in the parasternal short-axis papillary muscle level (PSAX-PML) view and processed through the ML algorithms so that they can extract the specific diagnostic features of the disease. This ML model surpassed the conventional methods with high accuracy, demonstrating ML’s ability to extract relevant data from imaging, such as ECHO, and provide a non-invasive approach for screening. If employed specifically to group 1 PH (PAH), these algorithms are capable of quickly detecting alterations in the pulmonary vasculature and PAP, thus providing the opportunity for early diagnosis and favorable results. Muriyama et al. used ML to analyze the ECHO PSAX-PML view with suspected cases confirmed with ECHOs and RHCs. The model extracted geometric features like ventricular area ratios and axis dimensions, showing a performance of AUC: 0.945 (internal) and 0.950 (external), and outperformed traditional assessments [132]. Similarly, various other studies have shown promising results using AI for analyzing the ECHO data, highlighting that ECHO can still remain the cornerstone of PH diagnosis and offer better screening than the conventional echocardiographic assessments [133]. To extend these benefits to SSc-PAH, such models must be trained using ECHO data from SSc patients, enabling the identification of subtle disease-specific patterns and improving early detection in this high-risk population.

#### 7.2.3. AI in CMR

CMR is another imaging modality that has an unparalleled ability to capture three-dimensional, high-resolution images of the heart and the vascular system, giving a detailed visualization of the cardiac anatomy, RV size, function, and pulmonary vascular changes, all of which are crucial in diagnosing PAH. Time-consuming and resource-intensive are two common issues related to traditional CMR metrics, leading to difficulties in incorporation in general clinical use. To tackle this problem, AI-enabled CMR interpretation models for the enhancement of the speed and accuracy of PAH detection are being used. Swift et al. introduced a tensor-based ML approach, a multilinear subspace learning AI model that achieved higher diagnostic accuracy, noted by an AUC at receiver operating characteristic (ROC) analysis (*p* < 0.001) of 0.92 for PAH as compared to the standard CMR metrics [134]. Moreover, the time taken by the model to establish a diagnosis was under 10 s, making it time-efficient. Additionally, whatever features the model learned were analyzed in the feature maps to visualize both the known diagnostic features as well as potentially new features linked to the pathophysiology of the PAH. AI-enhanced CMR models like these can give deeper insights into the cardiac remodeling and hemodynamic process, leading to early detection of the condition. According to the European Society of Cardiology/European Respiratory Society (ESC/ERS) guidelines, an accurate assessment of the RA area is crucial for predicting mortality in PH [51]. However, traditional manual measurements are prone to time constraints and inter-observer variability. To address this limitation, Alandejani et al. introduced a DLLGE-based RA area contouring model applied to CMR imaging [135]. Upon testing the model on a separate cohort of 400 patients, it showed higher intersubject repeatability with AI model measurements as compared to manual measurements, indicating very reliable and consistent measurements with the device. The model’s AI measurements of the RA area very closely matched the manual readings, aligned well with the invasive test results, such as RHC, and showed better accuracy at predicting elevated heart pressures. Therefore, AI-based RA size quantification can efficiently assess the RA, enhance the prognostic evaluation, and predict mortality risk. Wang et al. suggested a similar AI-assisted CMR interpretation model for screening and diagnosing 11 types of cardiovascular diseases, including PAH, which utilizes a two-stage approach [136]. To begin with, it entails a screening based on non-invasive cinematography, followed by a diagnosis based on cinematography and LGE. This AI model showed remarkable accuracy for diagnosis, even outperforming cardiologists in detecting PAH. Automated AI-driven CMR interpretation models, when integrated into routine screening, can be a promising addition to CMR protocols by improving early detection and risk stratification. This can especially be useful in conditions such as SSc-PAH, where cardiac involvement can progress immediately and requires the immediate attention of the clinicians.

#### 7.2.4. AI in CTPA

Like the PFTs, CTPA, when combined with AI, can also serve as a powerful non-invasive diagnostic tool. In patients suspected of having PH, Charters et al. showed that using an AI tool to calculate the RV/LV ratio from imaging can give us a strong idea if the right side of the heart is under any strain, which can be a clue for PH [137]. Normally, this ratio is calculated manually by the radiologists, but this AI tool had a higher sensitivity and strongly correlated with the RHC results as compared to the traditional methods. Though the research investigated PH in general, the RV/LV ratio is especially important for PAH (Group 1 PH), where RV dysfunction is a predominant characteristic. Furthermore, in their study, Zhang et al. presented an AI-based model for CTPA, which can non-invasively assess the PAP in patients with PH, and it was found to have a very good correlation with the RHC (ICC coefficient 0.93–0.98). Though the study encompassed mixed PH etiologies (21.8% were PAH cases), the model’s high performance in predicting PAP (AUC 0.91 for mPAP ≥ 40 mmHg) indicates possible clinical usefulness for managing PAH, especially in severity assessment and treatment monitoring; still, PAH-specific validation is required [138]. Having such AI-driven analysis in the routine interpretation of CTPA can allow clinicians to detect PAH in the early stages, even in centers that do not have access to invasive tests or an expert to read the cardiac imaging.

### 7.3. Multimodal AI Models for Comprehensive PAH Detection

Current approaches usually rely on single-modality data, which limits their ability to integrate crucial information such as medical images or reports into the system. This restricts their applicability in real-world clinical practice. With recent advances in clinical practice, there is an exponentially growing potential for combining data from multiple sources, making them more accurate and reliable. An early example of a multimodal approach, though not AI-based, is the predictive formula by Schreiber et al., which combined oximetry and PFT data to estimate mPAP [122]. Another one would be the DETECT algorithm that considers clinical symptoms, PFTs, ECGs, and TTE data for improved PAH detection in patients having SSc [82]. Taking this a step further, Zhao et al. proposed a comprehensive multimodal AI model that incorporates three major data types: tabular data (e.g., patient age, history, laboratory results), textual data (e.g., interpretation notes from ECHO, CXRs, and ECGs), and imaging data [110]. By combining different data sources, these multimodal models can bridge the current gap in the single modality approaches to improve diagnostic accuracy and prevent any unnecessary invasive procedure, such as RHC. These tools represent a significant advancement in making PAH detection and management more practical and scalable across various clinical settings. Table 1 compiles the studies described above [110,111,112,113,114,118,119,120,121,123,124,125,126,127,128,129,130,131,132,134,135,136,137,138], outlining the diagnostic modalities in which AI was applied, the specific tools and processes used, key advantages, outcomes, and the future potential of these approaches in the context of SSc-PAH.

Integrating AI into the conventional diagnostic modalities has started to revolutionize the detection of PAH by enhancing precision, reducing the need for invasive procedures, and allowing the real-time analysis of physiological, imaging, and clinical datasets. The progress made in this area is particularly important for SSc-PAH, which often shows minimal and vague early symptoms, and among the currently used screening tools, like TTE, DLCO, and NT-proBNP, they are all limited by the fact that they lack specific standalone sensitivity and specificity. AI’s demonstrated capability to detect hidden or subtle patterns in heart sounds [111,112], ECGs [119], CXRs [127,129], ECHO [130], and even EHRs [125] highlights its potential to revolutionize early, non-invasive risk stratification in this high-risk population. As this review will explore, these evolving AI applications not only highlight the feasibility of augmenting current tools but also provide a strong foundation for retooling and fine-tuning more robust, multimodal, and scalable diagnostic frameworks tailored to the complexities of SSc-PAH.

## 8. Existing AI Approach in Detecting SSc-PAH

AI approaches have been emerging for the detection of SSc-PAH to bring forward the early diagnosis and risk stratification. The achievement of early and accurate detection remains a difficult task in the field of medicine because of the vague and often subtle characteristics of SSc-PAH [139]. AI models, especially those employing ML and DL frameworks such as CNNs, are being widely utilized to bridge these gaps by discovering biomarkers, processing complex datasets, interpreting medical imaging more accurately, and so on. The focus of this segment is to investigate the research works that particularly highlight the ever-increasing role of AI in the early detection, diagnosis, and risk stratification of SSc-PAH, thus potentially benefiting the patients with better outcomes.

### 8.1. Genomics-Based Biomarker Identification

Xu et al. developed an AI-based diagnostic model to detect SSc-PH that focused on signal recognition particle (SRP)-related genes and their links to SSc-PH pathogenesis [139]. Gene expression data from public datasets were analyzed and found 30 differentially expressed SRP-related genes, all of which were downregulated in SSc-PH. Two ML algorithms were used: least absolute shrinkage and selection operator (LASSO) regression and support vector machine-recursive feature elimination (SVM-RFE), which identified and brought it down to seven key diagnostic genes (SRP-DGs). A scoring system (SRPscore) and a nomogram were constructed to quantify individual expression profiles, and an ANN was built using the SRP-DGs. It achieved a good diagnostic accuracy (AUC = 0.999 in training and 0.860 in testing). The study also found signaling pathways and immune characteristics associated with SRP dysfunction and identified ten potential drugs regulating these SRP-DGs. This demonstrated that AI can be integrated with genomics to identify reliable biomarkers for SSc-PH and provide early, precise, and personalized diagnosis. With further fine-tuning using SSc-PAH-specific data, this approach holds strong potential for improving the diagnosis of SSc-PAH.

### 8.2. CXR Image Analysis

Shimbo et al. employed a DL model to detect SSc-PH using chest X-rays [140]. The goal was to evaluate its performance compared to cardiologists, dermatologists, ECHO (TRV), and cardio-thoracic ratio (CTR). The model was constructed using the ResNet-50 architecture, which was pretrained on the cardiomegaly-labeled image dataset and fine-tuned using CXR and RHC data obtained from the reports of 230 SSc patients. It got an AUC of 0.826, having 82.6% accuracy, which was nearly the same as the assessments of cardiologists. The prediction score provided by AI was found to have a strong correlation with mPAP (r = 0.72, *p* < 0.001) and TRV (r = 0.74, *p* < 0.001) and was recognized as an important determinant of patient survival. Those who were found to be PH-positive using the model had a worse prognosis, confirmed by Kaplan-Meier survival analysis. This AI tool may aid early PH screening in SSc patients, especially where advanced diagnostics are unavailable. An especially important application of the tool is for the identification of SSc-PAH, the most common type of SSc-PH, and is the one most likely to be overlooked in the initial phases through the utilization of standard CXRs only. By detecting PAH without invasive methods and in resource-limited settings, the model could promote timely referrals, enable early management, and achieve better outcomes in SSc-PAH patients. The model’s performance relative to other modalities and clinical experts is summarized in Table 2 [140].

### 8.3. Echocardiographic Strain Pattern Detection

Preliminary results presented by Lui et al. (2024) demonstrated the potential use of AI in predicting SSc-PH [141]. Speckle tracking ECHO from 101 SSc patients was obtained, and researchers applied a random forest ML model to analyze regional myocardial longitudinal strain patterns in them. The system was able to competently identify precapillary PH through its cumulative performance of AUC: 0.805, accompanied by a good level of sensitivity of 0.79 and a specificity of 0.88. The investigators defined pre-capillary PH as mPAP > 20 mmHg, PAWP ≤ 15 mmHg, and PVR ≥ 3 Wood units, which aligned with the hemodynamic criteria in use at the time of the study, though slightly higher than the current ≥ 2 WU threshold recommended by the 2022 ESC/ERS guidelines [51]. This model therefore represents a valuable addition to SSc-PAH–focused approaches, offering a non-invasive tool for early risk stratification and for identifying patients who may benefit from confirmatory RHC.

### 8.4. Proteomics for Serum Biomarkers

According to a study conducted by Bauer et al. [142], the utilization of AI contributed to the improvement of detecting SSc-PAH at an early stage. The procedure was based on proteomics, and an RF algorithm was used on serum samples collected during the DETECT study, which was a famous program intended to identify early PAH in SSc patients. The application of ML to investigate the protein levels in blood samples of the DETECT cohort resulted in the identification of a group of 8 proteins that can non-invasively differentiate between SSc patients with or without PAH. Collagen IV, endostatin, IGFBP-2 (insulin-like growth factor binding protein), IGFBP-7, matrix metallopeptidase-2, neuropilin-1, NT-proBNP, and RAGE make up the eight-protein panel, mainly responsible for PAH pathogenesis. The panel in the DETECT group was shown to achieve an AUC of 0.741 with a sensitivity of 65.1% and a specificity of 69.0%. The findings were then validated through an independent Sheffield confirmatory cohort, where they reached a high AUC of 0.866. Furthermore, the sparse partial least squares (SPLS) regression was used to investigate the association among these biomarkers and the major PAH clinical markers. Several proteins, such as NT-proBNP, RAGE, and IGFBP-7, had a strong relationship with PVR, which is a main factor determining the level of disease severity. These results indicated that the AI-aided evaluation of high-dimensional proteomics data could be used even to analyze complex datasets of different protein levels and point to specific biomarkers that predict PAH in SSc patients, possibly allowing non-invasive screening before the onset of severe symptoms. The most significant advantage was the fact that the AI-led protein panel was better than NT-proBNP alone at identifying early PAH. Furthermore, the markers correlated with pathophysiological mechanistic processes, such as RV strain, endothelial injury, etc., thus demonstrating both diagnostic and prognostic applications by giving mechanistic insight into disease progression and monitoring.

### 8.5. Multimodal Clinical Data Integration

Lui et al. (2023) developed a multimodal (three prediction models) approach for diagnosing PH in SSc, integrating clinical and ECHO data [143]. The model used data from conventional diagnostic methods like PFTs, ECG, ECHO, and CT. The 3 models were RF, a classification and regression tree (CART) model, and a logistic regression model. The main intention of the authors was to evaluate the performance ability of the various models in the prediction of PH (confirmed by RHC). The model of the RF was the one that possessed the maximum accuracy of 88%, while the sensitivity and specificity were 95% and 80%, respectively. The logistic regression model featured quite similar accuracy, in contrast with the CART model, which had the worst performance. The major indicators for RF were the diameter of the pulmonary artery seen on a CT scan and the DLCO from PFTs, and these indicators were found to be better predictors than ECHO and ECG. Therefore, ML-based models built from regular, non-invasive diagnostic tests have the potential to assist clinicians in flagging patients requiring confirmatory RHC. Specifically, it can be further used for detecting SSc-PAH patients and to flag those who truly require RHC, enabling earlier and personalized treatment. The performance metrics of all three models are summarized in Table 3 [143], highlighting the superiority of ML methods in leveraging routine diagnostic data to identify patients who require confirmatory RHC.

### 8.6. Clinical Risk Factor Prediction

Yudkina et al. designed a simplified model to detect SSc patients who are at high risk of developing PAH [144]. The model was based on easily observable clinical signs and routine test results, like disease duration over 12 years, high uric acid levels, presence of telangiectasia, presence of anticentromere antibodies, and absence of antitopoisomerase-1 antibodies. The model was tested on 74 SSc patients undergoing their first RHC and was compared to existing screening algorithms like DETECT, ItinerAIR, and ASIG. The model demonstrated high sensitivity, negative predictive value (NPV), and accuracy. The key advantage of the model was its cost and simplicity, by using readily available parameters, thus avoiding the need for costly and invasive tools. The model was very good at identifying high-risk patients, thus helping clinicians flag at-risk PAH patients earlier and enabling better treatment outcomes. Although not AI-based, the simplified model developed by Yudkina et al. represents a clinically useful, low-cost decision tool built on easily obtainable parameters. Its high sensitivity and accuracy make it suitable for early risk stratification in resource-limited settings, with potential utility in guiding decisions about further invasive testing. Table 4 compares the performance metrics of the simplified model with existing screening algorithms [144].

### 8.7. Cluster-Based Mortality Prediction

Cerasuolo et al. evaluated the use of ML to predict 5-year cardiopulmonary mortality in SSc patients [145]. They demonstrated that the Partition Around Medoid (PAM) clustering method could effectively identify SSc patients at high risk of cardiopulmonary mortality, even if traditional risk factors are not completely present. They used the PAM clustering method on lab and functional parameters (such as FVC, DLCO, troponin, NT-proBNP, EF, and PAPs). The algorithm divided patients into two prognosis clusters with significantly different mortality rates: 15% vs. 0.8%. The high-risk group was found to have lower FVC and higher NT-proBNP and troponin levels. Paradoxically, a large part of the high-risk group did not show the classic clinical risk factors, such as being female, having a limited cutaneous involvement, or negative anti-Scl70; therefore, it is suggested that the AI be used to find not readily detectable hidden risk profiles instead of the traditional methods. The study stated that AI-based assessment of common cardiopulmonary data could serve as a useful instrument for both risk stratification and clinical decision-making in those situations where traditional forecasts might prove to be unreliable.

### 8.8. Early Functional Marker Identification

In a study by Koyama et al. [146], the researchers tried to focus on the subclinical stage of PAH, particularly exercise-induced pulmonary hypertension (exPH), as an early marker of disease progression. SSc patients who were at high risk due to clinical features like Raynaud’s phenomenon, skin sclerosis, or the presence of SSc-specific autoantibodies but had not yet developed overt PAH. Patients who were enrolled for six years underwent exercise Doppler ECHO (exDE) regularly to detect exPH, which is characterized by the increased systolic PAP (sPAP) during exercise. Peripheral blood RNA was isolated; the main markers were identified with machine learning (RF) after gene expression profiling. The discovered TNF (tumor necrosis factor) is a significant predictor of early disease development, with a model accuracy of 87%. This accuracy increased to 90% when the results were combined with TMEM176A/B gene expressions. The expression of TNF was found to be significantly higher only in the early (pre-exPH) stage of the disease, but the TMEM176A/B level was seen to be higher during all the stages of the disease. The results of the study are strong evidence of the role of AI in seeing the changes in the gene expression at the beginning, which others might miss, and show that SSc-PAH is a molecular disease with specific drivers of different biological markers. So, it can be suggested that AI is a potential instrument for early diagnosis of SSc-PAH and targeted phase-specific management.

### 8.9. Omics-Driven Risk Stratification

Launay et al. support a transference from the old-style algorithm approaches in SSc-PAH to precision medicine [147]. Through the incorporation of clinical data with omics like proteomics and transcriptomics, they envisioned AI playing a more secure and imperative role in SSc-PAH. The primary aim of the paper was to suggest non-supervised ML as a possibility for the consolidation of clinical data and biomarkers, with the ultimate effect on the accuracy of RHC decisions. The discovery of new biomarkers by ML, such as in the work of Bauer et al. [142], was also mentioned by the authors. Also, the authors expressed the possibility of the replacement or enhancement of the existing tools, like DETECT, by AI-driven tools. This AI-assisted method is very helpful in solving the problem of heterogeneity in SSc-PAH, provides optimal screening efficiency, and leads to the improvement of patients’ conditions through earlier interventions.

### 8.10. Serum Biomarker Differentiation

There was one particular research by Lemmers et al. that addressed the role of ML, especially the RF model, in the identification of serum biomarkers necessary to differentiate SSc with or without PAH [148]. Researchers studied 26 soluble serum biomarkers using RF that played an important role in identifying the top discriminative biomarkers. Among those, they named endostatin (elevated) and CXCL4 (decreased) as the best predictive biomarkers for SSc-PAH. The model was associated with an AUC of 0.92, which emphasized the model’s excellent ability to discriminate between SSc-PAH and SSc-noPAH. Hence, ML can facilitate both early diagnosis and biomarker identification, which further leads to the improvement of treatment outcomes in SSc-PAH. Table 5 presents the top-performing biomarkers along with their expression levels, statistical significance, and confidence intervals [148]. Table 6 summarizes the various key studies, outlining the AI/ML methods used, types of input data, main findings, model performance, and their clinical implications for improving SSc-PAH diagnosis and management [139,140,141,142,143,145,146,147,148].

To summarize, these emerging studies highlight the revolutionary potential of AI in the early detection and risk stratification of SSc-PAH. From analyzing imaging and clinical data to genomics and proteomics, AI models have demonstrated excellent performance across a spectrum of diagnostic tasks. Tools such as CNNs, SVM, RF, ANN, and multimodal learning approaches were found to be effective not only in detection but also gave insights into the underlying pathophysiology of SSc-PAH. For instance, AI has shown the ability to facilitate the identification of diagnostic genes, biomarkers, and subtle imaging features that are often overlooked by the human eye. Besides, simplified AI modules have been demonstrated to be effective not only as a screening tool but also as a cost-efficient and user-friendly option, particularly in resource-limited settings. Furthermore, these AI-driven models are proving effective for prognosis as well by flagging high-risk patients, thereby enabling individualized management plans. Despite their promise, however, most studies are limited to early-phase research and lack real-world clinical integration. At present, there is a pressing need for larger multi-institutional datasets, a series of strict prospective trials, and the formulation of related ethical guidelines to enable clinicians to incorporate AI in their day-to-day operations. However, the intersection of SSc-PAH-specific data and the progressive development in AI makes it possible for AI to be used as a strong modality to achieve significant reduction in diagnostic delays, prompt appropriate referrals, and consequently, better outcomes for patients suffering from this threatening complication.

## 9. Discussion

SSc-PAH is a severe and life-threatening complication of SSc [8] and presents with diagnostic challenges due to its insidious onset, complex pathogenesis, and nonspecific symptoms [139,149,150]. AI has emerged as a powerful aid in revolutionizing the landscape of SSc-PAH by leveraging multimodal data to allow earlier detection, enhance risk stratification, and enable individualized treatment approaches [143]. While novel AI solutions hold potential, there is also an equally significant and often overlooked prospect of the thorough reconfiguration of the existing AI models, especially those that were developed for PAH and general SSc, to remedy the specific diagnostic requirements of SSc-PAH. In a broader PAH context, AI is already being utilized to analyze traditional diagnostic methods like ECHOs, CMRs, heart sounds, biomarkers, etc. [113,118,132,136]. Still, the models need to be further calibrated with SSc-PAH-specific data. We propose a framework of a supervised learning algorithm system that will be retrained on a wider population of SSc-PAH patients, which will include not only the standard cardiopulmonary inputs like NT-proBNP, DLCO, TR velocity, and RV size but also autoimmune markers (e.g., anti-centromere, anti-U1 RNP), digital microvascular findings, and skin fibrosis scores. CNNs can be utilized on the unprocessed ECGs or the CXR scans, while omics layers like transcriptomic or proteomic data present the possibility of integrative depth for systems-level learning [121,128,147]. One of the fundamental features of a longitudinal model architecture is that it can regulate the thresholds dynamically based on patient outcomes and progression of disease. A conceptual minimal viable feature set for a multimodal AI model could integrate key clinical (e.g., FVC%/DLco%, autoantibodies), imaging (e.g., AI-quantified RA area), and biomarker (e.g., NT-proBNP, endostatin) data. An intermediate-fusion architecture, combining features extracted by separate modality-specific networks, presents a pragmatic initial approach. Prospective validation would necessitate a head-to-head comparison against DETECT/ASIG in an SSc cohort, using RHC-confirmed diagnosis as the gold standard to evaluate practical clinical benefit. This kind of AI system would not replace traditional diagnostics, but it will function as a triage filter, flagging individuals with the highest risk for early referral to RHC or specialist assessment. Concurrently, the few existing SSc-PAH-specific AI models that are available should receive additional development work and should be externally validated instead of being simply discarded. A pragmatic integration strategy would involve embedding AI models at defined points within existing screening algorithms like DETECT and ASIG. In DETECT, AI could be applied at Step 1 (pre-ECHO triage) to integrate clinical, serologic, and physiologic variables and generate a refined risk score, safely excluding low-risk patients from further testing and reducing unnecessary Step 2 evaluations. At Step 2 (ECHO augmentation), AI-enabled echocardiographic analysis (e.g., automated TRV or RA area quantification) could improve measurement reproducibility and detect subtle abnormalities. An AI-derived probability score could then be layered on top of the existing TRV/RA criteria to refine referral decisions. For ASIG, AI could act post-screen by combining NT-proBNP and PFT inputs with additional multimodal features (e.g., proteomics, ECG strain patterns) to generate a continuous probability estimate of PAH, rather than a binary threshold. In both algorithms, the goal would be to maintain or increase sensitivity (minimizing missed SSc-PAH cases) while stabilizing RHC referral volumes by lowering false-positive referrals. Conceptually, one could set the AI threshold to preserve the current RHC referral rate while capturing additional true positives that might otherwise be overlooked. Prospective studies will be required to define optimal cutoffs, benchmarked against DETECT/ASIG, using net reclassification and decision-curve analyses to quantify clinical utility. Acknowledging the severity and subtlety of SSc-PAH and its non-specific symptoms, often overlapping with other differential diagnoses, makes it crucial to modify and retool the well-established AI frameworks rather than developing from the ground up. This would likely unveil the most practical and meaningful in the proposed trajectory. We acknowledge that this proposed pathway is conceptual rather than operational. At present, we did not specify features, fusion strategies, thresholds, or deployment schema, as such details require future research supported by large-scale, multiethnic datasets and rigorous validation frameworks. The purpose of presenting this conceptual framework is to highlight the potential of multimodal AI approaches in SSc-PAH and to encourage collaborative efforts toward developing and testing operational models.

### 9.1. Advancing Diagnosis and Disease Classification

AI-based models have demonstrated strength in enhancing the diagnostic precision of SSc-PAH by identifying subtle or hidden patterns, often overlooked by traditional interpretations. DL algorithms trained on CXRs, ECHOs, ECGs, and CMRs have outperformed conventional modalities in detecting early signs of PAH [119,129,130,135]. For instance, CNNs applied to clinical data can detect RV strain and pulmonary artery dilation with notable sensitivity [137]. Moreover, AI tools can help differentiate SSc-PAH from differential diagnoses such as ILD or left heart disease. Innovative techniques like phonocardiogram analysis, ML interpretation of auscultation sounds, and wearables also hold potential for non-invasive, quick, point-of-care screening, especially in resource-limited settings [112,113,124]. AI’s capability to uncover hidden signals from multiple diagnostic methods is a game changer, allowing the restructuring of the diagnostic criteria and the adoption of wider and earlier screening strategies for systemic sclerosis populations.

### 9.2. Risk Stratification and Prognostic Applications

**Figure 2:**
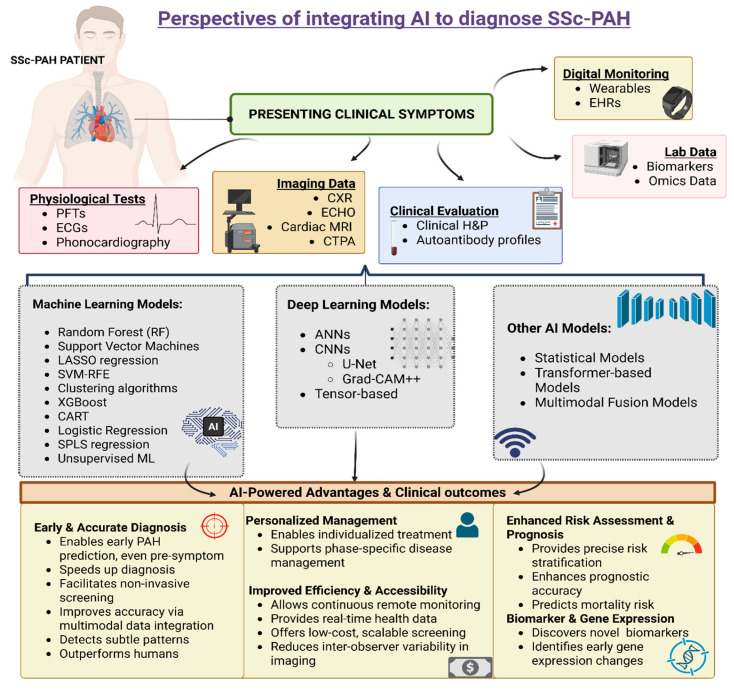
Perspectives of Integrating AI to Diagnose SSc-PAH.

ML and RF trained on clinical, proteomic, genomic, and imaging data have shown the capability to predict outcomes such as need for hospitalization, mortality, and RV failure. Tools that combine data from CTPA, PFTs, and ECHOs can find patients who require urgent RHC and prevent unnecessary invasive tests in low-risk cases. The proteomic AI models devised by Bauer et al. stand out for their dual purpose of not only making an early diagnosis of PAH but also giving directions on treatment and prognosis, which, in turn, paves the way for real-time management [141,142,143,145]. Figure 2 illustrates a comprehensive framework detailing the integration of AI for the diagnosis and management of SSc-PAH, highlighting the diverse data inputs, AI model types, and the anticipated clinical advantages.

### 9.3. Challenges and Limitations

Although AI-driven models have shown great strides in various applications, certain limitations impede their widespread clinical translation. The shortcomings of numerous AI studies, such as small sample sizes and limited generalizability to single-center or demographically narrow datasets, are evident. Besides, the heterogeneity of SSc itself, together with the inconsistency in labelling patients with a diagnosis (e.g., RHC-confirmed versus ECHO-suspected cases), adds yet another layer of complexity to the model training and evaluation processes. Many of the included studies are retrospective, relying on tertiary care datasets enriched with high-risk patients. This introduces selection bias, as models trained in such settings may overestimate predictive performance and fail to generalize to community or low-risk populations where early detection is most needed. Furthermore, AI models must account for heterogeneity in age, sex, ethnicity, and disease subtype. Failure to stratify across demographic subgroups risks propagating algorithmic bias, where predictive accuracy may be high for the training cohort but significantly lower in underrepresented populations. Ensuring equitable performance requires validation across multiethnic, sex-balanced datasets. Another significant hurdle is the interpretability of AI algorithms. Models that are simply black boxes and do not offer sufficient rationale for their results may be confronted with distrust by clinicians, which hinders the integration into their workflows. Therefore, transparent, comprehensible AI models are needed. Ethical questions related to data privacy, algorithmic bias, and regulatory oversight also remain pressing issues, which require the formulation of strong frameworks to take responsible action in development and deployment [151,152,153,154,155,156,157,158,159,160,161,162,163].

### 9.4. Future Directions

The future of AI in SSc-PAH is bright, yet it is contingent upon sustained innovation and rigorous validation. Prospective studies should focus on the following [163]:

1. Detailed Model Validation: To train and externally validate the models, we will need larger, multiethnic, and prospective datasets. Collaboration within international SSc-PAH registries and consortia (e.g., EUSTAR, PHAROS) can enhance model generalizability and reduce bias. Approaches such as federated learning, which allow multi-institutional training without raw data exchange, may help overcome data governance barriers while preserving privacy. Performance metrics such as the sensitivity, specificity, PPV, and NPV should be clearly defined and divided into subgroups (e.g., early disease versus advanced disease) to improve clinical relevance. Rigorous external validation in independent, multiethnic cohorts is non-negotiable to mitigate site effects, ILD confounding, and spectrum bias before clinical deployment. Overfitting must be proactively addressed through techniques like LASSO or ridge regression, dropout layers in deep learning, and systematic validation strategies.
2. Enhanced Data Integration: The combination of genomic, proteomic, imaging, and EHR data, with the advancement of AI, facilitates the process of deep phenotyping. With the adoption of this strategy, it may be possible to identify new subtypes of SSc-PAH, providing mechanistic insights and refining clinical classifications beyond the current frameworks.
3. Evaluation Beyond AUC: Future studies developing AI models for SSc-PAH screening must move beyond reporting the area under the curve (AUC) alone. To demonstrate tangible clinical benefit, performance should be benchmarked against current standards like the DETECT algorithm using decision-curve analysis to assess clinical utility across risk thresholds and net reclassification improvement to quantify the correct reclassification of patients.
4. Shift to Non-Invasive Methods: The ongoing improvements in AI models could achieve the preclusion or decrease in the need for invasive methods such as RHC, thus laying the foundation for the possibility of monitoring with complete non-invasiveness using PFTs, imaging, and biochemical markers over the long term.
5. End-Stage Disease Applications: AI could help the specialists make decisions on mechanical circulatory support or advanced therapies when they must deal with severe complications such as heart failure.
6. Seamless AI Integration in Healthcare: To achieve clinical success, the existing models need to be smoothly incorporated into EHRs and must give interpretable outputs that support, rather than take the place of, the clinician’s judgment. AI literacy training programs for health workers are also necessary to develop trust and to ensure proper use.
7. Adherence to Reporting Standards: The current landscape of AI studies in SSc-PAH is characterized by limited adherence to emerging guidelines such as TRIPOD-AI and CONSORT-AI, reflecting their retrospective, early-phase nature. As the field progresses, future prospective model development and validation studies must commit to these standards to ensure transparency, reproducibility, and critical appraisal of methodological quality.
8. Ethical and regulatory considerations: The initiation of AI devices in actual clinical practice makes it very critical to come up with the regulatory frameworks that address the issues of data privacy, algorithmic fairness, liability, and accountability. Ethical deployment is necessary for the preservation of public trust.

## 10. Conclusions

SSc-PAH is one of the most serious complications of SSc, with high morbidity and mortality rates. It is characterized by its unique traits, such as multifaceted features and insidious onset. Although significant progress has been made in both the detection techniques and screening protocols, the elusive problem at the clinical level remains the timely and accurate diagnosis. This often results in management delays and unfavorable treatment outcomes. AI offers an innovative approach to overcoming these diagnostic and prognostic barriers. Using ML, DL, and imaging analysis technologies along with the power of AI, one can analyze a data set that is complex, large, and multimodal, like PFTs, biomarkers, genes, and imaging. AI has shown notable performance in reducing diagnostic delays, enhancing prognostic accuracy, refining risk assessment, supporting non-invasive screening, and facilitating personalized management strategies. Different applications like interpreting nailfold capillaroscopy, quantifying pulmonary fibrosis, and predicting organ involvement in SSc show the potential of AI models in the management of SSc. However, the integration of AI into routine clinical practice needs to address certain major limitations and challenges, including data variability, model transparency, lack of previous validation, and ethical concerns based on algorithmic biases and patients’ rights. Fine-tuning AI tools specifically for the SSc-PAH population through robust, large multicenter datasets and disease-specific training holds potential for achieving clinically impactful outcomes. Importantly, future progress will depend on collaborative international consortia such as EUSTAR and PHAROS, as well as the application of federated learning approaches to overcome data governance and generalizability challenges. Rigorous external validation in independent, multi-ethnic cohorts will also be essential to ensure clinical applicability and equity in access.

In conclusion, the implementation of AI in the diagnosis and risk stratification of SSc-PAH has the potential to transform disease management by enabling earlier detection, accurate risk assessment, and individualized treatment planning. Continued research, interdisciplinary collaboration, and ethical deployment of these technologies will be essential to achieving this goal and improving patient care.

## Figures and Tables

**Table 1:** AI applications in various traditional diagnostic modalities for PAH with potential relevance to SSc-PAH.

Study Author	Year	Modality	AI- Technology	Process	Outcomes	Advantages	Future Potential of AI in SSc-PAH
Charters et al. [137]	2022	CTPA	ResNet-18 + SegNet (CNN)	Automated RV/LV ratio calculation using CT images	ICC: 0.878 (vs. manual RV/LV), AUROC: 0.752 for PH detection, Sensitivity 73%, Specificity 67% (threshold ≥ 1.12)	Reduces inter-observer variability (manual ICC 0.791–0.928); Stronger correlation with RHC; Predicts mortality; Screening in suspected PH	Combine CTPA with PFTs/ECG for multimodal screening of suspected SSc-PAH.
Zhang et al. [138]	2023	CTPA	3D nnU-Net + XGBoost	Extracted 1D/2D metrics (e.g., LV diameter, RAd/LAd ratio) and correlated with RHC-derived PAP	ICC (vs. RHC mPAP): 0.934, AUC for classification: mPAP ≥ 40 mmHg: 0.911	Non-invasive PAP estimation; High accuracy in severe PH detection (mPAP ≥ 40 mmHg)	Extend to detect early PAH in SSc with fine-tuned metrics.
Kwon et al. [119]	2020	ECG	DL	Wave pattern analysis (S/P/T waves)	Early PAH prediction even before symptom onset	Superior to human interpretation; Low-cost; scalable; Useful in resource-limited settings	PAH-specific fine-tuning using SSc patient data.
Liu et al. [120]	2022	ECG	DL	Evaluated 10 AI models using 12-lead ECGs paired with ECHO estimates of PAP)	AUC: 0.88; Sensitivity: 81%, Specificity: 80%, Accuracy: ~80%	Identify patients at risk before clinical PH develops	Can flag high-risk SSc patients for follow-up before symptoms appear.
DuBrock et al. [121]	2024	ECG	DL-CNNs	Developed PH Early Detection Algorithm (PH-EDA) trained on ECGs of PH and non-PH patients.	AUC 0.92 (internal), 0.88 (external); long-term predictive accuracy	Outperforms human interpretation; works on normal-appearing ECGs	Train PH-EDA on SSc cohorts to identify asymptomatic early SSc-PAH cases.
Zou et al. [129]	2020	CXR	DL	Classifies PH vs. normal CXR	AUC up to 0.945; Accuracy 86.14%	Better than subjective human CXR review	Train to separate SSc-PAH from other PH types for targeted management.
Imai et al. [128]	2024	CXR	CNNs	Classifies PAH vs. normal CXR	sensitivity of 93.3%, specificity of 98.2%, and an AUC of 0.988	Early, non-invasive detection with high diagnostic accuracy	Fine-tune using CXR datasets from SSc patients with and without PAH.
Kusunose et al. [127]	2022	CXR	DL	Analyzes pre- and post-exercise CXRs	82% accuracy	Identifies early vascular changes; No need for stress ECHO; Widely accessible	Combine with EHR data for resting PAH prediction.
Rasmy et al. [125]	2021	EHR	Med-BERT (BERT-based NLP model)	EHR data analysis (ICD codes)	Improved AUC by 1.21–6.14% in disease prediction tasks	Processes large datasets very efficiently; Real-time alerts.	Adapt for early, EHR-based detection of SSc-PAH using structured codes + temporal trends in symptoms and visits; integrate with imaging/ECG models for a multimodal SSc-PAH diagnostic tool.
Schuler et al. [126]	2022	EHR	ML	EHR data analysis (ICD codes, CPT codes and medications)	Sensitivity: 88%, Specificity: 93%, Positive Predictive Value (PPV): 89%, Negative Predictive Value (NPV): 92%	Reducing need for manual chart reviews; scalable; facilitates early identification of PAH	PAH-specific fine-tuning using SSc patient data.
Elgendi et al. [113]	2018	Heart Sounds	Linear Discriminant Analysis (LDA)	Extracted heart sound features (e.g., frequency band power, entropy) and trained on PAH vs. non-PAH data	Sensitivity: 84%, Specificity: 88.57%	Low-cost, non-invasive, interpretable model using standard auscultation	Can be adapted for screening SSc-PAH in remote settings; potential for telehealth integration.
Wang et al. [114]	2022	Heart Sounds	Transfer Learning (ResNet101, DenseNet201)	Used pretrained CNNs to classify PCG recordings into six heart condition categories, including PH	Accuracy: 90–98% for various heart conditions	Robust against noise; generalizable to real-world recordings	Can be developed into mobile-based screening tools in SSc with high specificity.
Ogawa et al. [111]	2024	Heart Sounds	Super Stethoscope + AI	Visual heart sound mapping from ECHO + auscultation	Detects elevated pressures, murmurs	Provides quantitative data vs. subjective auscultation	Use in remote areas for early SSc-PAH flagging from heart sound patterns.
Guo et al. [112]	2025	Heart Sounds	DL Phonocardiogram (spectrogram-based)	Trained on >170,000 PCGs with PASP/ECHO labels; mel-spectrogram input	Sensitivity: 0.71, Specificity: 0.73	Captures inaudible clues missed by physicians	Deploy for community-level SSc-PAH screening in resource-poor or remote settings.
Lewis et al. [118]	2020	Biomarkers	ML	NT-proBNP + clinical variable analysis	Personalized prediction and risk stratification	Combines lab and clinical context unlike static cutoffs	Develop point-of-care NT-proBNP + AI platforms for SSc-PAH monitoring.
Salehi et al. [130]	2025	ECHO	US2.AI (DL) model	Auto-calculates TR jet velocity	High concordance with RHC; 87% interpretability	Faster and less operator-dependent than manual echo	Use in clinics for early screening of RV pressure in SSc.
Liao et al. [131]	2023	ECHO	ML	PSAX-PML ECHO + ML classification	AUC 0.945 in the internal validation (vs. 0.892, *p* = 0.027), and 0.950 in the external validation.	Detects subtle ECHO features	Train on SSc-specific ECHO features to detect early vasculopathy.
Muriyama et al. [132]	2024	ECHO	ML	Extracted geometric features from 346 ECHOs with RHC confirmation	AUC: 0.945 (internal), 0.950 (external)	Outperformed traditional methods	Fine-tune with SSc data for early, accurate detection.
Swift et al. [134]	2021	CMR	ML(tensor model)	Feature extraction from cine images	AUC: 0.92; diagnosis time < 10 s	Detects hidden imaging patterns missed in manual CMR reads	Apply to SSc-specific cardiac remodeling patterns.
Alandejani et al. [135]	2022	CMR	DL	RA area contouring	Accurate, consistent RA area measures	Reduces inter-observer variability; matches RHC data	Predict mortality and monitor remodeling in SSc-PAH follow-up.
Wang et al. [136]	2024	CMR	Two-stage AI model (cine + LGE)	Cine + LGE-based disease classification	Outperforms cardiologists for PAH	More efficient than manual reading; wider disease coverage	Identify SSc-PAH progression patterns with fibrosis insight.
Hughes et al. [123]	2025	Wearables	Fitbit + ML	Tracks sleep, activity trends	Decreased REM/light sleep + steps in PAH	Real-world tracking vs. snapshot tests like 6MWT	Incorporate trends into AI models for early decline in SSc-PAH.
Hesar et al. [124]	2023	Wearables	ML + EES	Seismocardiogram + ECG via smartphone	Extracts prognostic index in real time	Continuous, mobile monitoring outside hospital	Add to telehealth platforms for SSc-PAH symptom monitoring.
Zhao et al. [110]	2025	Multimodal	Transformer Model	Tabular + text + imaging fusion	AUC 0.96 for PAH prediction	Integrates multiple data sources and superior to single modality	Deploy as a real-time clinical decision support tool.

**Table 2:** Performance comparison of the ResNet-50 deep learning model for SSc-PH detection on CXRs versus standard diagnostic methods and clinical expert assessments.

Method	AUC	Sensitivity	Specificity
AI	0.826	50.00%	94.10%
ECHO (TRV)	0.927	58.30%	100%
CTR	0.786	66.70%	79%
CARDIOLOGISTS	0.804	~70%	~67%
DERMATOLOGISTS	0.756	~57%	~71%

**Table 3:** Performance comparison of three predictive models for PH diagnosis in systemic sclerosis using multimodal clinical and imaging data.

Model	Accuracy	Sensitivity	Specificity
RF	88%	95%	80%
Logistic Regression	78%	85%	70%
CART	63%	55%	70%

**Table 4:** Comparison of performance metrics of the simplified PAH screening model and existing algorithms in systemic sclerosis.

Algorithm	Sensitivity (%)	Specificity (%)	PPV (%)	NPV (%)	Accuracy (%)
DETECT	100	26	44	100	53
ASIG	74	55	49	79	62
ItinerAIR	100	28	56	100	54
Simplified model	96	70	65	97	80

**Table 5:** Key serum biomarkers differentiating SSc-PAH from SSc-noPAH identified using a RF Model.

Biomarker	Level in SSc-PAH	*p*-Value	95% CI of Difference
Endostatin	↑ Increased	0.004	16 [5 to 27] pg/mL
sVCAM1	↑ Increased	0.04	47 [2 to 92] pg/mL
VEGF-D	↑ Increased	0.03	205 [18 to 391] pg/mL
CXCL4 (PF4)	↓ Decreased	0.0002	−1963 [−2956 to −970] pg/mL
sVEGFR2	↓ Decreased	0.0009	−3258 [−5135 to −1381] pg/mL
PDGF-AB/BB	↓ Decreased	0.013	−3870 [−6913 to −826] pg/mL

**Table 6:** Summary of AI applications for diagnosis, risk stratification, and prognosis in SSc-PAH.

Study Author	Study Year	AI Method	Input Data	Key Findings	Performance	Clinical Implications
Xu et al. [139]	2022	LASSO, SVM-RFE, ANN	Gene expression data focusing on (SRP)-related genes	Identified 30 downregulated SRP-related genes in SSc-PH; narrowed down to 7 key diagnostic genes (SRP-DGs); constructed SRPscore and nomogram; developed ANN for diagnosis	AUC: 0.999 (training), 0.860 (testing)	Demonstrated potential of integrating AI with genomics for early and precise diagnosis of SSc-PH; identified signaling pathways and immune characteristics associated with SRP dysfunction; proposed 10 potential drugs regulating SRP-DGs.
Shimbo et al. [140]	2024	DL model using ResNet-50 architecture	CXRs and RHC data from 230 SSc patients	Detected SSc-PH using CXRs; compared performance with cardiologists, dermatologists, ECHO (TRV), and CTR	AUC: 0.826; Accuracy: 82.6%; Sensitivity: 50.0%; Specificity: 94.1%	AI model’s prediction score correlated strongly with mPAP (r = 0.72) and TRV (r = 0.74), a significant predictor of patient survival and a potential tool for early PAH screening in SSc patients, especially in resource-limited settings.
Lui et al. [141]	2024	RF	Speckle tracking ECHO data from 101 SSc patients	Predicted pre-capillary PH; analyzed regional myocardial longitudinal strain patterns	AUC: 0.805; Sensitivity: 79%; Specificity: 88%	Provided a non-invasive method for early risk stratification and identification of patients who may benefit from confirmatory RHC; aligned with current hemodynamic criteria for SSc-PAH.
Bauer et al. [142]	2021	RF; SPLS regression	Proteomic analysis of serum samples from the DETECT study	Identified an 8-protein panel to distinguish between SSc patients with and without PAH	AUC: 0.741 (DETECT cohort); AUC: 0.866 (Sheffield validation cohort); Sensitivity: 65.1%; Specificity: 69.0%	Demonstrated that AI-based analysis of proteomic data can identify specific biomarkers predictive of PAH in SSc patients; potential for earlier, non-invasive screening before severe symptoms onset.
Lui et al. [143]	2023	RF, CART, Logistic Regression	Combining PFTs, ECG, ECHO, and CT images data	Developed three prediction models for diagnosing PH in SSc; compared the performance of the models	Random Forest performed the best: Accuracy: 88%; Sensitivity: 95%; Specificity: 80%;	Highlighted that ML-based models built from regular, non-invasive diagnostic tests can assist clinicians in flagging patients requiring confirmatory RHC; potential for early and personalized treatment of SSc-PAH.
Cerasuolo et al. [145]	2023	PAM clustering	Laboratory and functional parameters such as FVC, DLCO, troponin, NT-proBNP, EF, and PAPs	Identified SSc patients at high risk of 5-year cardiopulmonary mortality; divided patients into two prognosis clusters with significantly different mortality rates	Mortality rates: High-risk group: 15%; Low-risk group: 0.8%	Demonstrated that AI-driven analysis of routine cardiopulmonary data can be a valuable tool in risk stratification and clinical decision-making, especially in areas where conventional predictors might fail.
Koyama et al. [146]	2024	Random Forest	exDE and peripheral blood gene expression profiling	Focused on the subclinical stage of PAH, particularly exPH; identified TNF as the strongest predictor of early disease progression; combined with TMEM176A/B gene expressions for improved accuracy	Model accuracy: 87%; Increased to 90% when combined with TMEM176A/B expressions	Highlighted the importance of AI in detecting early, subtle, complex patterns in gene expression that conventional methods may miss; revealed that SSc-PAH develops in distinct molecular phases, enabling targeted phase-specific management.
Launay et al. [147]	2021	Unsupervised ML	Integration of clinical data with omics, such as proteomics and transcriptomics	Highlighted the potential of non-supervised ML to combine clinical data and biomarkers to improve the accuracy of RHC decisions	Not specified	Suggested that AI-driven tools could eventually replace or enhance current tools like DETECT; aimed to address heterogeneity in SSc-PAH, optimize screening efficiency, and improve patient outcomes through earlier interventions.
Lemmers et al. [148]	2023	RF	Analysis of 26 soluble serum biomarkers	Identified Endostatin (elevated) and CXCL4 (decreased) as the most predictive biomarkers to distinguish SSc with or without PAH	AUC: 0.92	Demonstrated that AI can enable early diagnosis and biomarker identification in SSc-PAH, improving treatment outcomes; emphasized the potential of serum biomarkers in non-invasive screening.

## Data Availability

The raw data supporting the conclusions of this article will be made available by the authors on request.

## Abbreviations

The following abbreviations are used in this manuscript:

AI	Artificial Intelligence
ML	Machine Learning
DL	Deep Learning
ANN	Artificial Neural Network
CNN	Convolutional Neural Network
SVM	Support Vector Machine
EHR	Electronic Health Records
CXR	Chest X-Ray
CTPA	CT Pulmonary Angiography
CMR	Cardiac Magnetic Resonance Imaging
ECHO	Echocardiogram
TTE	Transthoracic Echocardiography
RHC	Right Heart Catheterization
mPAP	Mean Pulmonary Arterial Pressure
PAWP	Pulmonary Artery Wedge Pressure
PVR	Pulmonary Vascular Resistance
PASP	Pulmonary Artery Systolic Pressure
TRV	Tricuspid Regurgitation Velocity
PH	Pulmonary Hypertension
PAH	Pulmonary Arterial Hypertension
SSc	Systemic Sclerosis
lcSSc	Limited cutaneous SSc
dcSSc	Diffuse cutaneous SSc
ILD	Interstitial Lung Disease
DLCO	Diffusing Capacity of the Lung for Carbon Monoxide
PFT	Pulmonary Function Test
FVC	Forced Vital Capacity
ASIG	Australian Scleroderma Interest Group
6MWT	Six-Minute Walk Test
VO_2_ peak	Peak Oxygen Uptake
VE/VCO_2_	Minute Ventilation to Carbon Dioxide Output Ratio
V/Q	Ventilation/Perfusion
